# Long-Term Kinetics of Serological Antibodies against *Vibrio cholerae* Following a Clinical Cholera Case: A Systematic Review and Meta-Analysis

**DOI:** 10.3390/ijerph19127141

**Published:** 2022-06-10

**Authors:** Basilua Andre Muzembo, Kei Kitahara, Debmalya Mitra, Ayumu Ohno, Shin-Ichi Miyoshi

**Affiliations:** 1 Graduate School of Medicine, Dentistry and Pharmaceutical Sciences, Okayama University, Okayama 700-8530, Japan; keikitahara@okayama-u.ac.jp (K.K.); py386nyz@okayama-u.ac.jp (A.O.); miyos-s@okayama-u.ac.jp (S.-I.M.); 2 Collaborative Research Center of Okayama University for Infectious Diseases in India, Kolkata 700010, India; debmalya889@gmail.com

**Keywords:** cholera, antibodies, vibriocidal, cholera toxin B, lipopolysaccharide, immunoglobulin, immunity, waning

## Abstract

Background: Approximately 2.9 million people worldwide suffer from cholera each year, many of whom are destitute. However, understanding of immunity against cholera is still limited. Several studies have reported the duration of antibodies following cholera; however, systematic reviews including a quantitative synthesis are lacking. Objective: To meta-analyze cohort studies that have evaluated vibriocidal, cholera toxin B subunit (CTB), and lipopolysaccharide (LPS) antibody levels following a clinical cholera case. Methods: Design: Systematic review and meta-analysis. We searched PubMed and Web of science for studies assessing antibodies against *Vibrio cholerae* in cohorts of patients with clinical cholera. Two authors independently extracted data and assessed the quality of included studies. Random effects models were used to pool antibody titers in adults and older children (aged ≥ 6 years). In sensitivity analysis, studies reporting data on young children (2–5 years) were included. Results: Nine studies met our inclusion criteria for systematic review and seven for meta-analysis. The pooled mean of vibriocidal antibody titers in adults and older children (aged ≥ 6 years) was 123 on day 2 post-symptom onset, which sharply increased on day 7 (pooled mean = 6956) and gradually waned to 2247 on day 30, 578 on day 90, and 177 on day 360. Anti-CTB IgA antibodies also peaked on day 7 (pooled mean = 49), followed by a rapid decrease on day 30 (pooled mean = 21), and further declined on day 90 (pooled mean = 10), after which it plateaued from day 180 (pooled mean = 8) to 360 (pooled mean = 6). Similarly, anti-CTB IgG antibodies peaked in early convalescence between days 7 (pooled mean = 65) and 30 (pooled mean = 69), then gradually waned on days 90 (pooled mean = 42) and 180 (pooled mean = 30) and returned to baseline on day 360 (pooled mean = 24). Anti-LPS IgA antibodies peaked on day 7 (pooled mean = 124), gradually declined on day 30 (pooled mean = 44), which persisted until day 360 (pooled mean = 10). Anti LPS IgG antibodies peaked on day 7 (pooled mean = 94). Thereafter, they decreased on day 30 (pooled mean = 85), and dropped further on days 90 (pooled mean = 51) and 180 (pooled mean = 47), and returned to baseline on day 360 (pooled mean = 32). Sensitivity analysis including data from young children (aged 2–5 years) showed very similar findings as in the primary analysis. Conclusions: This study confirms that serological antibody (vibriocidal, CTB, and LPS) titers return to baseline levels within 1 year following clinical cholera, i.e., before the protective immunity against subsequent cholera wanes. However, this decay should not be interpreted as waning immunity because immunity conferred by cholera against subsequent disease lasts 3–10 years. Our study provides evidence for surveillance strategies and future research on vaccines and also demonstrates the need for further studies to improve our understanding of immunity against cholera.

## 1. Introduction

Cholera is extremely contagious and has a significant impact on public health [1] and can thereby negatively impact the economy [2]. It is an acute dehydrating diarrheal disease caused by the Gram-negative bacterium, *Vibrio cholerae*. The *V. cholerae* species includes >200 serogroups, two of which (O1 and 139) are mainly responsible for cholera outbreaks or epidemics [2,3]. *V. cholerae* O1 is further classified into Ogawa and Inaba serotypes [3]. This bacterium is found in fresh water, estuarine and brackish environments (its ecological niches), either floating freely or attached to aquatic flora and fauna, including phytoplankton and zooplankton [4,5]. It is transmitted to humans through consumption of contaminated food or water or direct contact with infected feces (such as by touching infected fomites) [6]. Moreover, it infects humans using two virulence factors, namely toxin-coregulated pilus and cholera toxin, which both play a key role in the occurrence of diarrhea [7].

Cholera was first recognized around the Ganges Delta and had spread worldwide, which has caused seven pandemics during the nineteenth and twentieth centuries [2,8]. The ongoing seventh pandemic is caused by *V. cholerae* O1 El Tor biotype strains and appears to have emerged from Indonesia in 1961 [9]. Despite centuries of efforts to control cholera, it continues to be endemic in >50 countries, such as in the Indian subcontinent and Sub-Saharan Africa [6].

Cholera affects people of all ages in endemic areas; however, young children bear the greatest disease burden [10]. The risk of cholera is especially high in destitute communities burdened by challenges, such as poor sanitation and limited access to safe water, poor health systems, and lack of infrastructure [6,11,12]. The risk of cholera is also high during humanitarian crises. For instance, cholera outbreaks have been reported among Rwandan refugees [13], in countries experiencing civil unrest such as Yemen [14] and the Democratic Republic of the Congo [15], and in Haiti after an earthquake [16].

*V. cholerae* causes approximately 2.9 million cases of cholera per year worldwide, resulting in approximately 95,000 deaths (between 21,000 and 143,000) [8,17]. Several deaths during cholera outbreaks or epidemics can be attributed to a lack of adequate preparedness [14,18,19]. Patients suffering from severe cholera can die within hours following the symptom onset due to dehydration and hypovolemic shock [8]. Fortunately, with timely treatment and appropriate case management with oral rehydration salts or intravenous rehydration, mortality occurs only in <1% of patients with cholera [8,20]. However, cholera can be considered as a neglected disease and remains among the leading causes of morbidity and mortality, even though the disease and death can be averted by socioeconomic development, mass availability of oral cholera vaccines, and targeted use of these vaccines [12,21].

To highlight the significant interest in fighting against cholera, in October 2017, the World Health Organization (WHO) Global Task Force on Cholera Control (GTFCC) launched a roadmap to fight cholera [11]. The GTFCC primarily aimed to reduce cholera deaths by 90% worldwide. It also aims to eliminate cholera by 2030 in at least 20 countries with emphasis on multi-sectoral interventions, including access to safe water, adequate sanitation, and hygiene and research [11]. Furthermore, the WHO recommends the use of cholera vaccines as adjuncts to fight against cholera [11,21,22].

Research has demonstrated that studying the protection level conferred through natural cholera infections is one way of estimating the protection cholera vaccines might provide [22]. Therefore, understanding the duration of serological antibodies conferred by natural cholera is vital when designing studies on vaccine development, immunization guidelines, and surveillance efforts [23]. Furthermore, serological studies on cholera can provide convincing evidence for pandemic preparedness [24].

Exposure to *V. cholerae* triggers the production of the serum vibriocidal antibody and other antibodies directed against specific antigens, such as cholera toxin B subunit (CTB) and lipopolysaccharide (LPS) [25].

Cholera anti-CTB and anti-LPS antibodies have been reportedly associated with the protection against *V. cholerae* infection [26]. Anti-CTB antibodies are believed to prevent cholera by binding to *V. cholerae* toxins, whereas anti-LPS antibodies prevent cholera by inhibiting *V. cholerae* from adhering and colonizing the gut [8,27].

Furthermore, vibriocidal responses are also associated with protection against *V. cholerae* infection. Previous studies have demonstrated that anti-CTB and anti-LPS antibodies and vibriocidal antibody titers sharply increase immediately (within 7–30 days) after symptom onset and rapidly decrease within 360 days [28,29]. Following clinical cholera, serum anti-CTB and anti-LPS antibodies provide a better indication of immune protection than the vibriocidal antibody [30]. Several cohort studies reported the expression of antibodies following cholera, and several reviews on this topic have been published [10,25,28,31]; however, none of them have carried out a meta-analysis, i.e., the evidence has not been quantitatively synthesized yet. Therefore, we conducted a systematic review and meta-analysis to evaluate how antibody levels change quantitatively over time after clinical cholera.

This study brings attention to a data gap in other countries; for instance, we found that most studies on long-term serological antibodies against *V. cholerae* were conducted in only one country, Bangladesh. Thus, this study also provides clinicians, policymakers, and global health agencies with additional quantitative information about waning antibodies following cholera.

## 2. Methods

Study design: A systematic review and meta-analysis was conducted following the guidance from the Preferred Reporting Items for a Systematic Review and Meta-analysis (PRISMA) [32] and registered in the international prospective register of systematic reviews (PROSPERO; registration number: CRD42022324892).

### 2.1. Data Sources and Searches

PubMed and Web of Science were searched for related studies from inception to December 2021. This study used the same search strategy as used in Leung and Matrajt’s systematic review [25]. Search strings combined Medical Subject Heading terms and free terms. For cholera, the following keywords were used: “Cholera” OR “*Vibrio cholerae*” OR “*Vibrio cholerae* O1”. The keywords above were combined with the following immunity-related keywords: “immunity” OR “immune” OR “immunologic” OR “antibody”. Our searches were further refined by adding the following terms: “vibriocidal”, “toxin B subunit”, “lipopolysaccharide”, and “memory B cell”. We also manually searched the reference lists of selected studies and related key reviews.

We used Endnote software X9 (Clarivate, Philadelphia, PA, USA) to manage the retrieved citations (such as removing duplicate references).

### 2.2. Study Selection

Studies had to meet the following criteria to be eligible for inclusion: (1) the study must have been performed on humans with clinically confirmed cholera (population); (2) the study must have assessed exposure to *V. cholerae* O1 or O139 (exposure); (3) a study without mandatory comparison group (comparison); (4) the study must have assessed changes over time in antibody responses to *V. cholerae* O1 or O139 (outcomes); and (5) cohort studies with at least 3 months of follow-up) (study design).

We excluded cross-sectional studies and those failing to meet the minimum inclusion criteria (for instance, studies carried out on animals, those conducted exclusively on vaccinees and with <3 months of follow-up, those available only in abstract format, letters, editorials, review articles, and commentaries). First, two investigators (BAM and KK) independently screened the titles and abstracts of the retrieved studies. Following that, full texts of potentially relevant studies were retrieved and screened for inclusion. Reasons for exclusion were recorded, and disagreements were resolved through discussion.

### 2.3. Data Extraction and Quality Assessment

To extract data, a data extraction sheet using Microsoft Excel 2019 (Version 2204, Microsoft Corp., Albuquerque, NM, USA) was designed. Data were extracted by two investigators. Any disagreements were resolved through consensus, and further reading of the articles. Extracted data included author names, year of publication and study period, setting, sample size, design, age of study population, antibodies measured and their measurement methods, and follow-up duration. All data regarding antibody levels were extracted from figures using the WebPlotDigitizer tool (Version: 4.5, Ankit Rohatgi, Pacifica, CA, USA). Moreover, data extraction from figures was also performed by a private company (Statista Consultants, Kyoto, Japan) to ensure accuracy.

Two investigators independently evaluated the quality of studies using the Newcastle-Ottawa Scale (NOS) for cohort studies [33]. A third investigator (AO) was consulted in cases of any disagreement.

### 2.4. Data Synthesis and Analysis

Meta-analysis was performed using Stata software (version 16, StataCorp LP, College Station, TX, USA). Furthermore, Microsoft Excel 2019 (Version 2204, Microsoft Corp., Albuquerque, NM, USA) was also used to generate bar graphs. Random-effect models were used to account for heterogeneity that frequently occurs in meta-analysis. We calculated the pooled mean of antibody levels and the corresponding 95% confidence intervals (CIs) at different time points (acute phase and during convalescence). Data from studies that had evaluated more than one group of patients were all considered data points. Sensitivity analysis was also performed to assess the impact on pooled data estimates that included children aged 5 years or younger. We assessed heterogeneity using Cochran’s Q and I^2^ statistics. Cochran’s Q with *p* < 0.1 and I^2^ of >50% were deemed to indicate substantial heterogeneity [34].

Tables, graphs, and forest plots were used to present antibody kinetic results. Moreover, textual narratives were used to report the remaining results. All data on the pooled mean are presented with their 95% CIs in parenthesis.

## 3. Results

### 3.1. Search Results

Supplementary Figure S1 displays the literature search and selection summary. We retrieved 4703 records from electronic databases and 15 through manual search. Of these, 727 duplicates and 3915 were excluded based on their titles and abstracts, leaving 76 records evaluated in full. Out of 76 records assessed in full, 67 were excluded because they did not meet our inclusion criteria. Studies were excluded mainly because of their shorter follow-up duration. Thus, nine studies met our inclusion criteria [35,36,37,38,39,40,41,42,43], and seven were used in the meta-analysis [35,37,38,39,40,42,43].

### 3.2. Study Characteristics

Characteristics and details of the nine included studies are presented in Appendix A Table A1. All of them were published in English between 2008 and 2019. The sample sizes ranged from 14 to 320 participants. Samples were collected at various time points. In most studies, longitudinal antibody assays were performed from day 2 to 360. However, only one study [35] performed blood collection until day 900. Most studies included patients with *V. cholerae* O1 (both Inaba and Ogawa serotypes).

All patients with cholera were from one cholera-endemic country (Bangladesh); however, two were challenge studies from the United States of America [35,36]. Four studies clearly stated that they had included patients with severe dehydrating cholera [40,41,43,44]. Most studies were conducted on adults and older children (aged ≥ 6 years). However, two studies included adults and children aged ≥ 2 years [35,37]. In two studies, vaccinees were used as controls [38,39]. The included studies assessed immune markers, including vibriocidal antibodies, anti-CTB IgA, anti-CTB IgG, anti-LPS IgA, and anti-LPS IgG in cohorts of patients with clinical cholera. All studies measured blood antibodies. However, one study also measured mucosal antibodies [40]. All studies used the same method to measure vibriocidal titers using guinea pig complement (Appendix A Table A1). Conversely, enzyme-linked immunosorbent assays (ELISA) were used to measure anti-CTB IgA, anti-CTB IgG, anti-LPS IgA, and anti-LPS IgG.

The blood group has been suggested to play a role in susceptibility to cholera. Six studies [35,36,37,38,39,40] used the blood group, whereas three others did not provide relevant information [41,42,43]. A summary of the methodological assessment of the included studies is displayed in Appendix A Table A2. The quality of reporting was satisfactory in most studies. Scores ranged between 5 and 8 out of 9. Four studies scored 8.

### 3.3. Antibody Kinetics

Findings from primary studies were consistent, showing that anti-CTB IgA and anti-CTB IgG levels, anti-LPS IgA and anti-LPS IgG, and vibriocidal titers are relatively higher during early convalescence (i.e., on days 7 and 30) compared to day 2 post-symptom onset and then they gradually decline.

One longitudinal study evaluating serological antibodies found that during the immediate convalescent phase (i.e., at the 7th and 30th days), anti-CTB IgA and IgG increased, but not anti-CTB IgM [41]. Furthermore, the same study reported anti-LPS IgA and anti-LPS IgG and anti-LPS IgM increased on days 7 and 30 post-symptom onset [41].

One study demonstrated that long-term immunity following cholera might not be mediated by mucosal antibodies found in the gut constitutive discharge [40]. Their findings were illustrated by the relatively short duration of antibody expression at the surface of the gut mucosa compared to blood during the convalescence phase. The same study found that anti-LPS IgA and anti-LPS IgG antibody levels increased in duodenal extracts on day 30, but their levels waned off on day 180 [40]. Similarly, the peak of mucosal anti-CTB antibodies occurred on day 30; however, these antibodies were statistically significant only for anti-CTB IgG antibodies [40].

### 3.4. Meta-Analysis Results by Antibody Types

The following five *V. cholerae*-specific antibodies were included in the meta-analysis: vibriocidal titers, anti-CTB IgA, anti-CTB IgG, anti-LPS IgA, and anti-LPS IgG.

#### 3.4.1. Vibriocidal Antibody Titers

**Figure 1:**
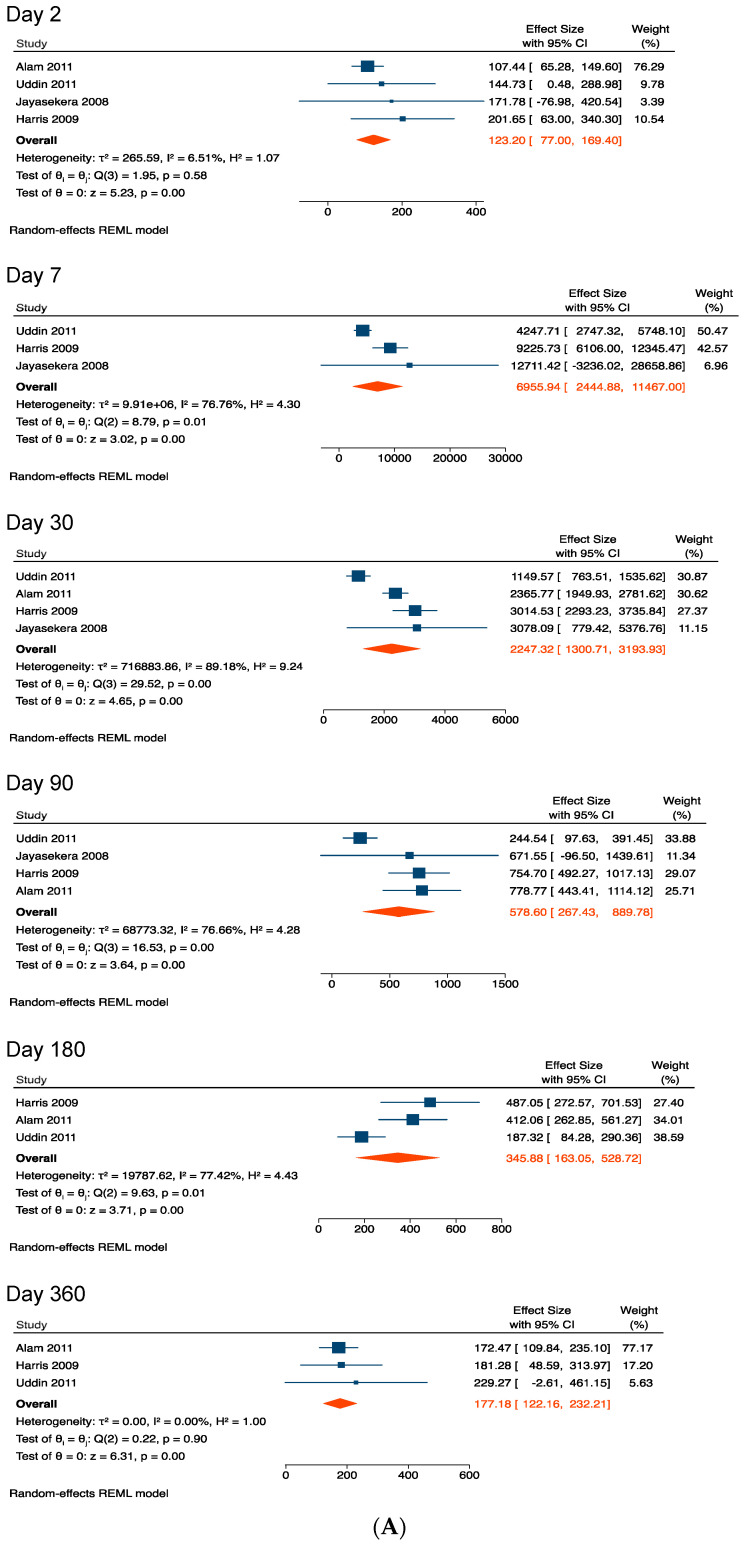

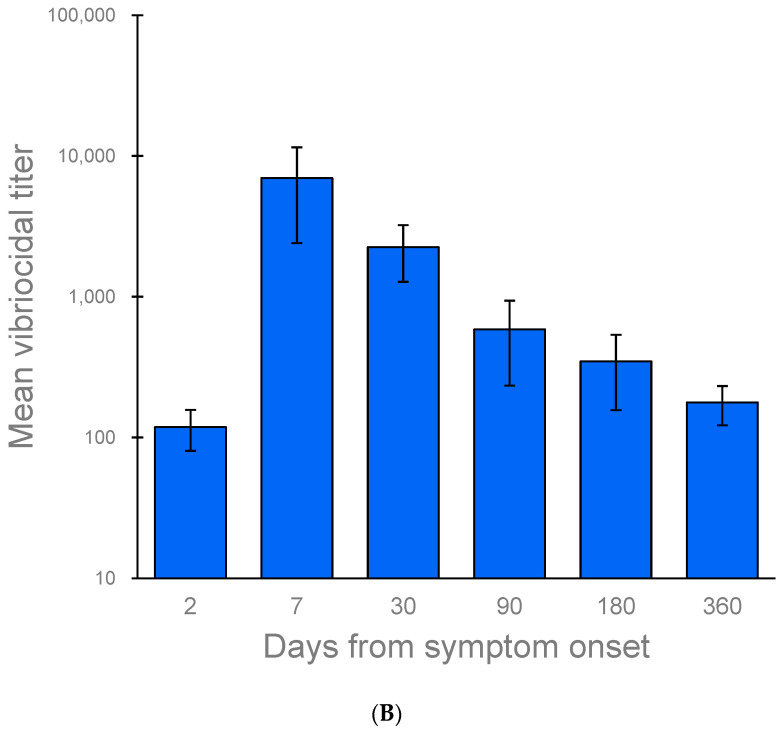
(**A**) Forest plots of the kinetics of vibriocidal antibody titers after cholera [37,39,40,42,43]. (**B**) Kinetics of vibriocidal antibody titers after cholera.

The pooled mean vibriocidal antibody titer for adults and older children (aged ≥ 6 years) on day 2 from symptom onset was 123.2. Vibriocidal antibody levels rapidly increased to reach a peak on day 7 (pooled mean = 6956.0), gradually waned to 2247.3 on day 30, and to 578.6 on day 90. Vibriocidal antibodies were still detectable on day 360 (pooled mean = 177.2) at levels higher than day 2 (Figure 1A,B). Two studies reported that vibriocidal titers on day 7 were higher in young children (aged 2–5 years) compared with those found for older children and adults [35,37]. Despite the fact that young children had the highest vibrocidal titers during the acute phase (day 7) [35], they returned to baseline on day 90, but remained elevated until day 180 in adults and older children during the convalescent period [37]. Sensitivity analysis was performed by including two studies that obtained data from young children [35,37]. We found that age has less influence on vibriocidal titers, and the pooled mean vibriocidal titers in the sensitivity analysis were comparable with those in the primary analysis (Table 1). Similarly, the sensitivity analysis revealed that the highest pooled mean vibriocidal titer was also observed on day 7 post-symptom onset and gradually decreased thereafter for >1 year after symptom onset (Table 1).

#### 3.4.2. Antibody Responses against the B Subunit of the Cholera Toxin

Anti-CTB IgA and IgG levels peaked between days 7 and 30 and then rapidly declined thereafter.

The pooled mean baseline anti-CTB IgA (mean of 5 on day 2 post-symptom onset) was lower in magnitude compared with that of anti-CTB IgG (mean of 18 on day 2 post-symptom onset).

**Figure 2:**
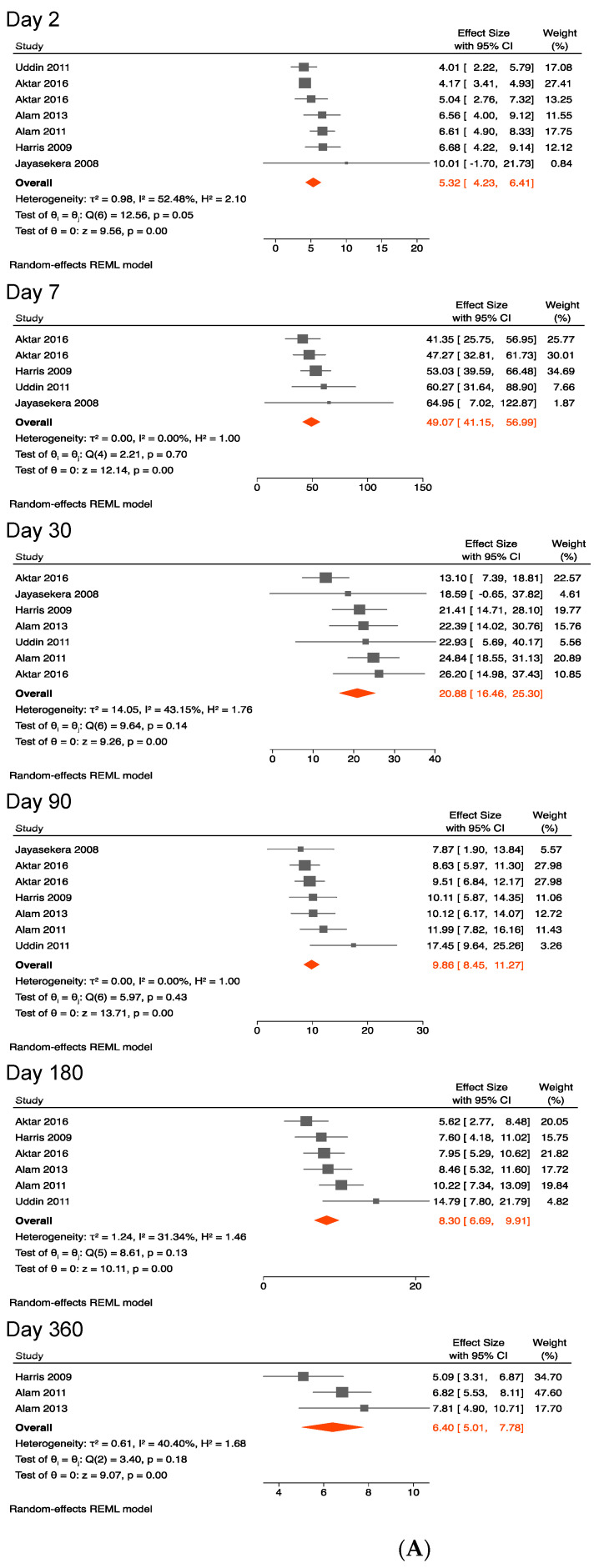

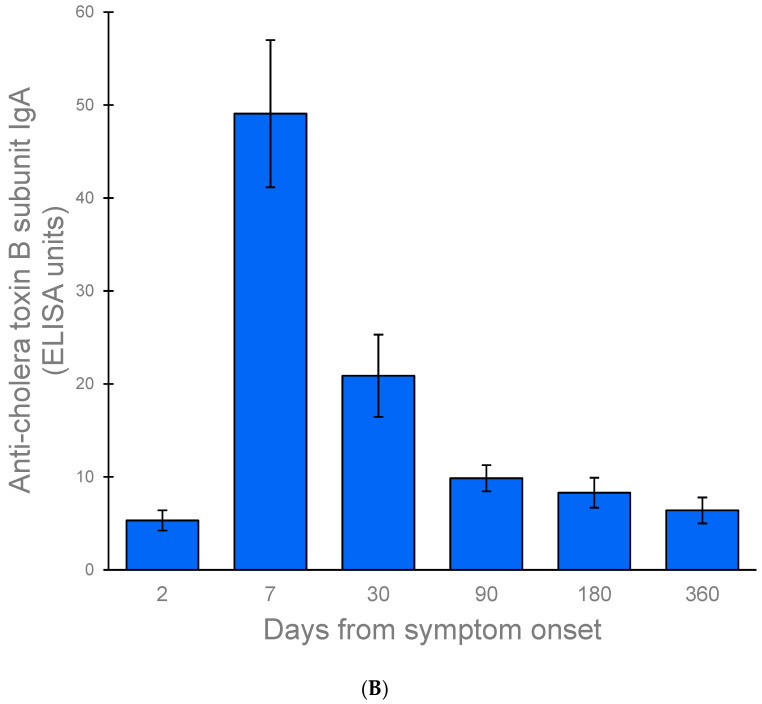
(**A**) Forest plots of the kinetics of anti-cholera toxin B subunit IgA following cholera [37,38,39,40,42,43]. (**B**) Kinetics of anti-cholera toxin B subunit IgA following cholera.

As shown in Figure 2A,B, anti-CTB IgA titers peaked on day 7 to a mean titer of 49 (41–57), decreased on day 30 to a mean titer of 21 (17–25), further waned to 10 (9–11) on day 90, and then plateaued from day 180 to 360.

On day 360, the anti-CTB IgA titer returned to levels comparable to those seen during an acute infection.

Sensitivity analysis, including two studies that contained data from young children (aged 2–5 years), showed very similar findings as in the primary analysis (Table 2).

**Figure 3:**
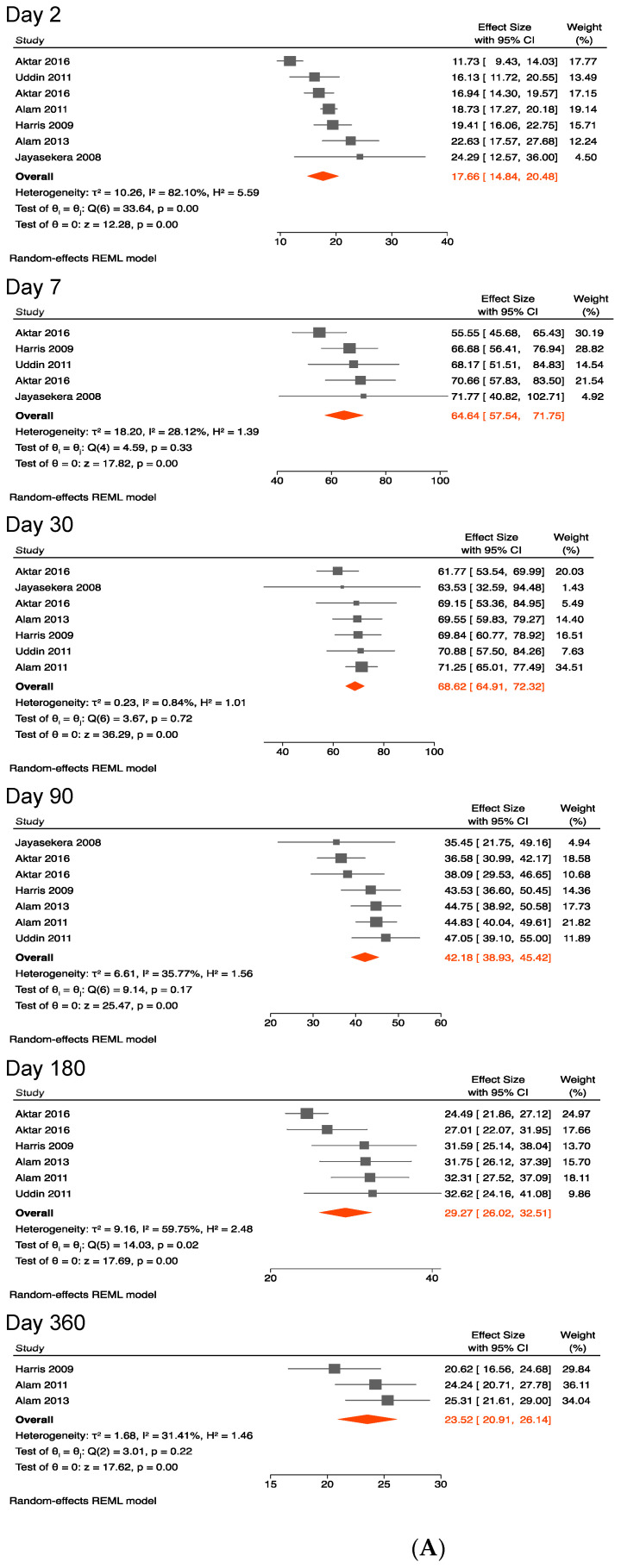

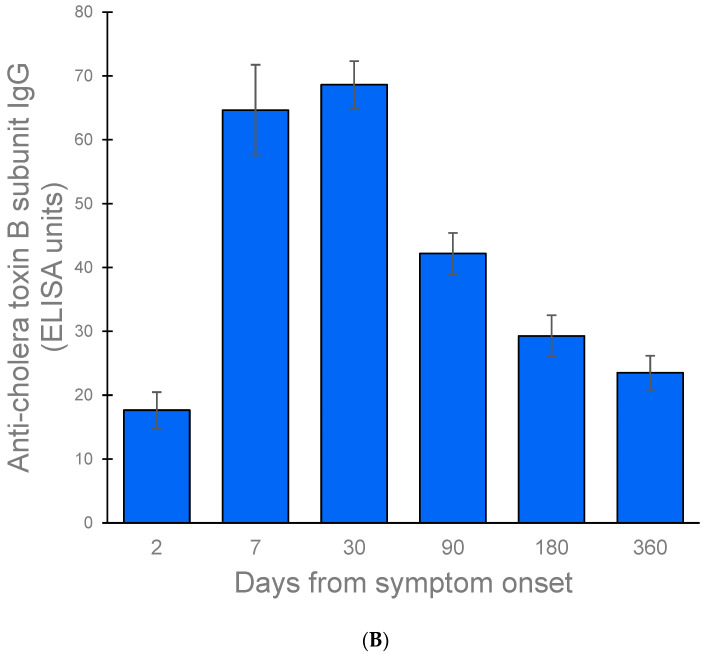
(**A**) Forest plots of the kinetics of anti-cholera toxin B subunit IgG following cholera [37,38,39,40,42,43]. (**B**) Kinetics of anti-cholera toxin B subunit IgG following cholera.

Similarly, anti-CTB IgG titers peaked at 65 (58–72) on day 7, at 69 (65–72) on day 30, then gradually waned to 42 (39–45) on day 90, and to 30 (26–33) on day 180, and then dropped to 24 (21–26) on day 360 (Figure 3A,B).

When compared with acute infection titers on day 2, anti-CTB IgG levels were still elevated 1 year after symptom onset.

The pooled mean anti-CTB IgG titers appeared to be comparable in the primary and sensitivity analyses including data from young children (Table 3).

#### 3.4.3. Antibody against Lipopolysaccharides of *V. cholerae* O1

We observed that the magnitude of IgG antibodies against LPS was greater than that of IgA antibodies.

**Figure 4:**
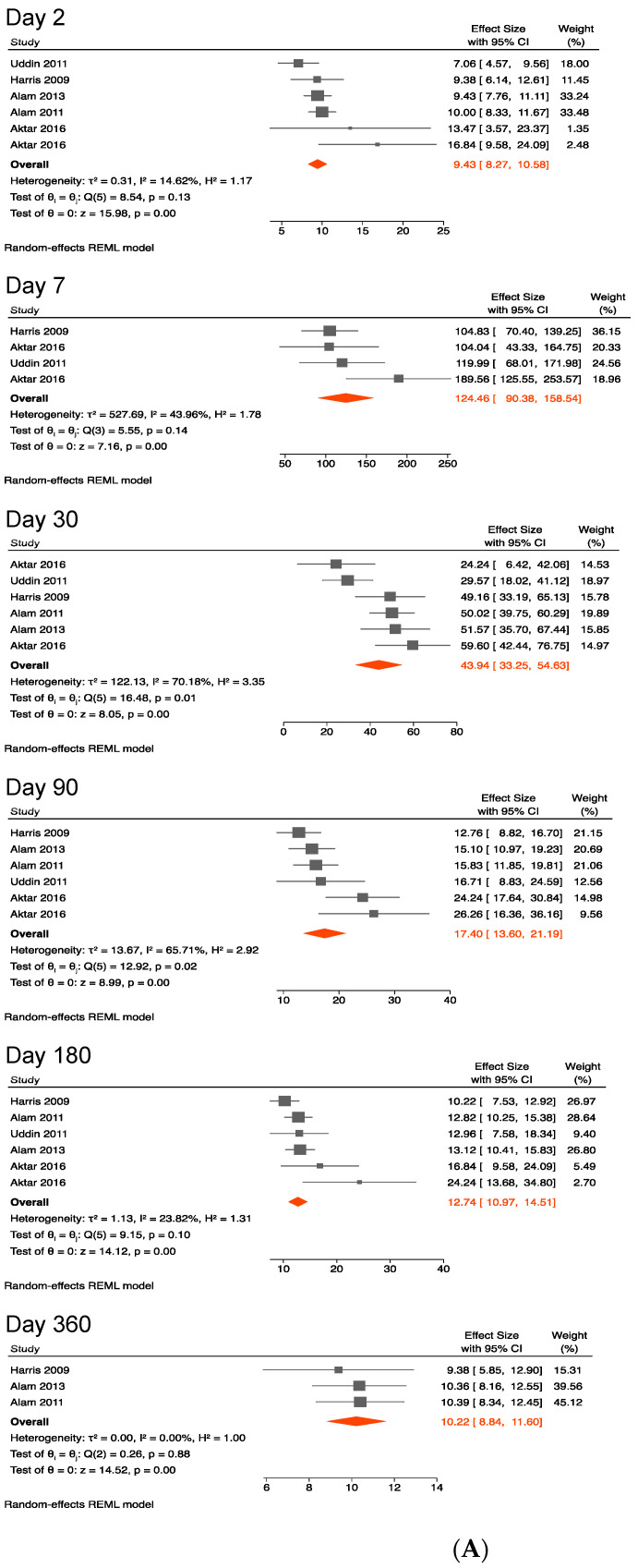

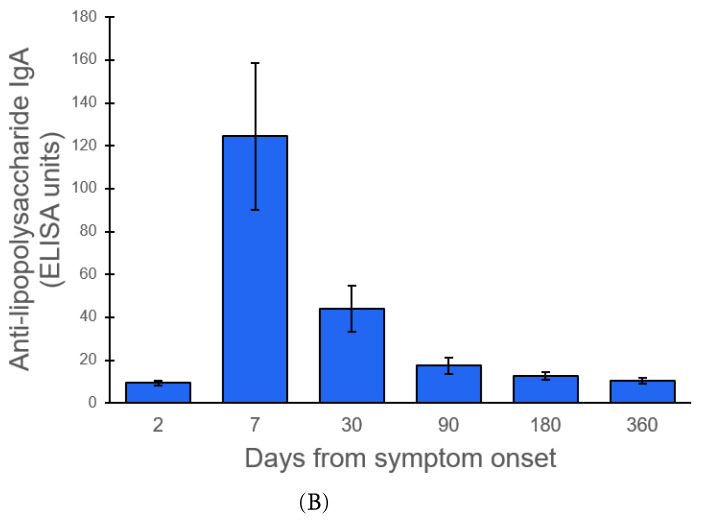
(**A**) Forest plots of the kinetics of anti-lipopolysaccharide IgA following cholera [37,38,39,40,42]. (**B**) Kinetics of anti-lipopolysaccharide IgA following cholera.

The pooled mean LPS-specific IgA antibody level was 9 (8–11) on day 2 following the symptom onset, peaked at 124 (90–159) on day 7 and gradually declined to 44 (33–55) on day 30, but persisted for at least 1 year. The pooled mean LPS-specific antibody IgA level was 10 (9–12) on day 360, a finding comparable to that on day 2 (Figure 4A,B). Sensitivity analysis revealed that the pooled mean did not change when including data from young children (Table 4).

**Figure 5:**
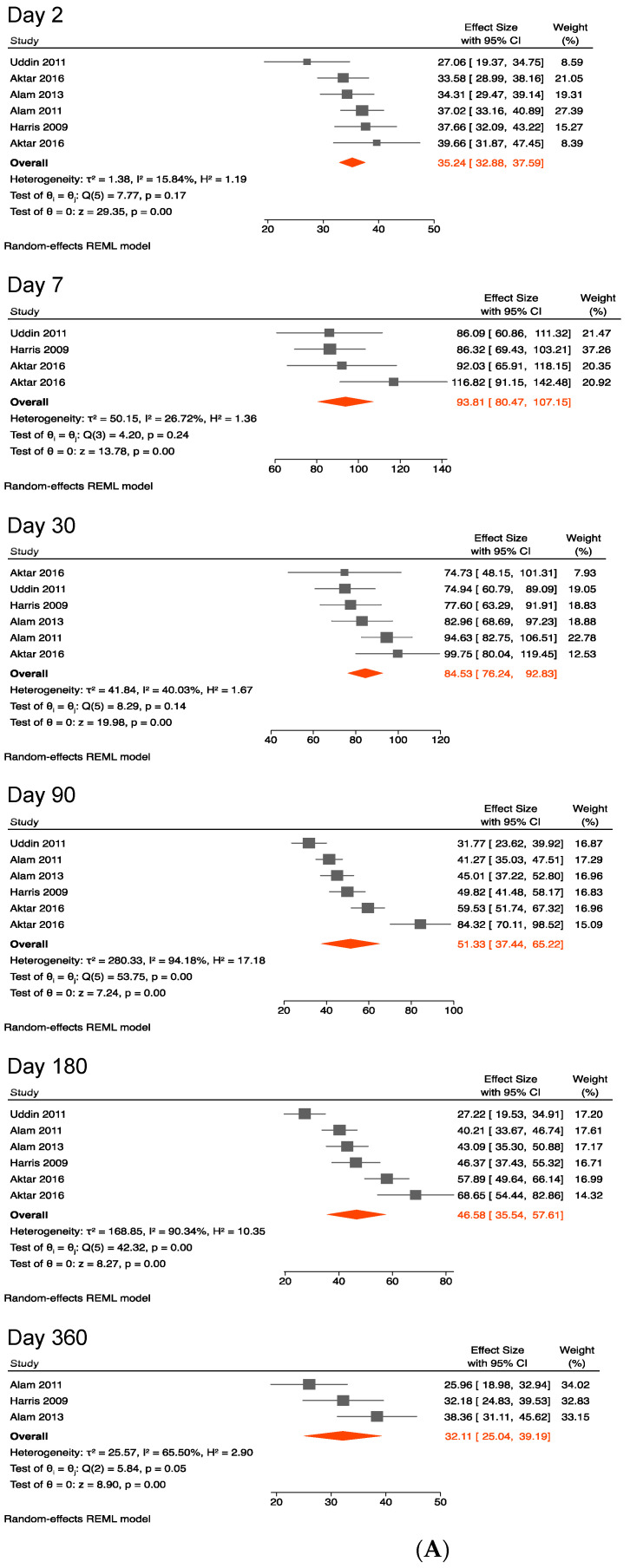

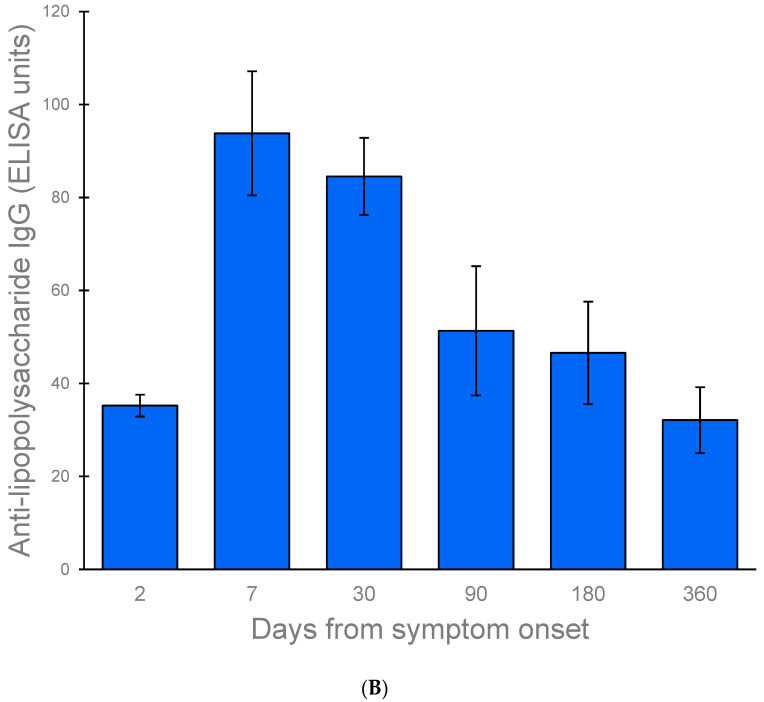
(**A**) Forest plots of the kinetics of anti-lipopolysaccharide IgG following cholera [37,38,39,40,42]. (**B**) Kinetics of anti-lipopolysaccharide IgG following cholera.

Figure 5A,B show that on day 2 following the symptom onset, the pooled mean LPS-specific IgG level was 35 (33–38). It followed a pattern in which it peaked and persisted at lower levels for >1 year. The pooled mean LPS-specific IgG antibody level gradually increased from 35 (33–38) on day 2 to 94 (81–107) on day 7. Thereafter, it decreased to 85 (76–93) on day 30 and further dropped to 51 (37–65) on day 90, to 47 (36–58) on day 180, and to 32 (25–39) on day 360. The pooled mean levels of LPS-specific IgG antibody remained unchanged when including two studies with data from younger children (Table 5).

Heterogeneity between studies was not excessive as shown by I^2^ values in Figure 1A, Figure 2A, Figure 3A, Figure 4A and Figure 5A.

## 4. Discussion

In this study, we synthesized the persistence of serological antibodies (vibriocidal, anti-CTB, and anti-LPS antibodies) in patients who had recovered from clinical cholera using cohort studies. All studies included in the meta-analysis were conducted from one cholera-endemic country, Bangladesh. This systematic review and meta-analysis confirmed that serum or plasma vibriocidal antibody titers, anti-CTB and anti-LPS antibodies return to baseline levels within 1 year following a clinical cholera case, i.e., before the protective immunity conferred by cholera against subsequent disease wanes (which lasts at least 3 years [22,45]). Given the fact that cholera vaccines function through antibodies and serological markers are used to evaluate related immune responses, this study partially fills our knowledge gap on evidence about the quantity and kinetics of serological antibodies following cholera. Remarkably, researchers focus on antibodies when they study adaptive immunity to cholera because antibodies are thought to mediate protection at the mucosal surface [31,46,47]. Thus, an understanding on the duration of serological antibodies after clinical cholera has critical implications in guiding preventive measures and vaccine research.

### 4.1. Vibriocidal Antibodies

Vibriocidal antibodies are bactericidal, complement-dependent serum antibodies produced by patients who had recovered from clinical or subclinical cholera infection [48]. Thus, vibriocidal titer assays measure the ability of serum antibodies to kill *V. cholerae* in the presence of complements [31].

Seroconversion occurs when vibriocidal titers increase fourfold or more compared to the baseline [49,50]. Our results revealed that the baseline vibriocidal titer (pooled mean of 123.20 (77.00, 169.40)) increased more than four times in the early convalescence (i.e., on day 7 post-symptom onset; pooled vibriocidal titers of 6955.9 (2444.9, 11,466.9)), suggesting seroconversion.

However, they gradually waned to the pooled vibriocidal titers of 177.2 (122.2, 232.2) during the late convalescence (i.e., on day 360 following the symptom onset), a level close to the baseline. Conversely, vibriocidal titers in vaccinees rapidly decayed to baseline within 360 days post-vaccination [20,35,51]. However, they can persist for >548 days (or >18 months) in patients who had recovered from cholera [35].

Since the protective immunity conferred by cholera against a subsequent disease can last at least 3–10 years [22,45], these data are unequivocal, suggesting that decay in vibriocidal antibody titers should not be translated directly into the waning immunity. Hence, vibriocidal titers alone cannot clearly explain the protection against reinfection. This observation supports the hypothesis that longer-term protective immunity might be mediated by other immune markers through anamnestic responses of memory B cells in the gut-associated lymphoid tissue [25,39,52].

Likewise, vibriocidal antibody titers are commonly used as indirect surrogate markers for longer-term immunity directed at the O-specific antigen of *V. cholerae* LPS [31,48]. Indeed, they are only a proxy for the intestinal mucosal immune status [8]. Although vibriocidal antibody titers are undoubtedly the best-accepted non-mechanistic correlate of protection against cholera, they are regarded as an imperfect marker for long-term immunity. Notably, no universally established threshold of vibriocidal antibody titers guarantees complete protection [2]. Furthermore, the correlation of vibriocidal antibody titers with protection remains debatable. Cholera vaccines that induce vibriocidal antibodies similar to those generated by wild-type infections have not clearly been clinically effective [28,53]. However, vaccine-induced vibriocidal seroconversion is associated with protection [51]. For instance, randomized controlled trials of cholera vaccines in cholera naïve populations demonstrated that an increase in vibriocidal antibody titers correlates well with protection against cholera [54]. Furthermore, studies conducted in Bangladesh found that vibriocidal antibody titers were significantly higher in uninfected household contacts of patients compared with both patients and contacts who subsequently became infected with *V. cholerae* [26,55]. Furthermore, contacts of uninfected patients were significantly older than those infected [26]. This is consistent with previous findings that vibriocidal titers increase with age, thereby decreasing the risk of severe disease in cholera-endemic areas [30,55,56]. Yet, another study reported that vibriocidal antibody titers in household contacts were equally associated with protection from infection regardless of age [57], suggesting that further investigations are needed. In our meta-analysis, the pooled vibriocidal titers were not influenced by age; however, since only two studies included data on younger children, our study might not have sufficient evidence for this outcome. The kinetics of serum vibriocidal antibodies is especially crucial because serum vibriocidal antibody titers are the most frequently used marker for evaluating vaccines [2].

### 4.2. Antibody Responses against the B Subunit of Cholera Toxins

Our meta-analysis revealed that anti-CTB IgA and IgG levels increased at least fourfold from the baseline between days 7 and 30. Although IgA level decreased more quickly over time, both IgA and IgG levels returned to baseline levels within 1 year. Comparing vaccinees and cholera convalescent patients, one study found that after cholera, anti-CTB IgA and IgG persist longer than after vaccination [38].

CTB-specific responses are T-cell-dependent (unlike LPS-specific responses, which are T-cell-independent) [38]. T cells have been hypothesized to be associated with activation and stability of memory B cells that withstand stimulatory cytokine release and crosstalk with cells in the lymph nodes, which in turn protect against subsequent cholera [26,52]. Despite the evidence that CTB is important for immunity, previous studies have yielded controversial conclusions regarding anti-CTB and protective immunity. Several previous studies have also noted no association between anti-CTB IgG and protection from cholera [26,30,58]. However, an association between anti-CTB IgA and protection from *V. cholerae* O1 infection was observed in household contacts of patients with cholera [30].

Moreover, research has demonstrated that CTB is nontoxic, which suggested that CTB possesses great immune modulation potential. Thus, CTB can be used in cholera vaccines as an antigen and in vaccines against several different diseases as a delivery molecule [59,60]. For example, the inactivated-whole-cell cholera vaccine (WC/rBS; Dukoral) is formulated with recombinant nontoxic CTB and is used to prevent cholera or travelers’ diarrhea. Anti-CTB responses in Dukoral are hypothesized to significantly contribute to extra short-term protection when compared to whole cell vaccines alone [61]. This observation is also consistent with the results of a phase 1 randomized controlled trial of the oral MucoRice-CTB vaccine, which reported that immunization with MucoRice-CTB induced high CTB-specific serum IgG and IgA levels [62].

### 4.3. Antibody against V. cholerae O1 Lipopolysaccharides

Both anti-LPS IgA and IgG peaked on day 7. However, they returned to baseline levels within 1 year, as in anti-CTB antibodies. Anti-LPS antibodies are known to be T-cell-independent [38]. They more efficiently enter the gut lumen and prevent *V. cholerae* from adhering and colonizing to the gut and might result in long-term protective immunity in individuals recovering from cholera [27]. Furthermore, clinical cholera leads to the development of anti-LPS antibodies with avidity indices that correlate with memory B cell responses; thus, protection against cholera is currently hypothesized to be mediated by anti-LPS antibodies and more specifically by antibodies for O-specific polysaccharides [28]. Indeed, consistent associations had been reported between OSP-specific IgA and IgG antibodies and a lower risk of infection by *V. cholerae* in household contacts of patients with cholera [63]. Moreover, high levels of LPS-specific IgA antibody secreting cells (ASCs) in lamina propria lymphocytes (LDL) have been observed in patients with cholera, denoting that LPS-specific IgA ASCs may persist over time [40]. However, patients who have recovered from cholera have longer-lasting avid anti-LPS IgA and IgG memory B cells than vaccinees [38], whose elevated levels of LPS-specific IgA and IgG memory B cells also correlate with vibriocidal antibody responses [51]. What is also striking is that although the data suggest that anti-LPS IgA and anti-CTB IgA are associated with cholera immunity, these antibodies may not be long-term mediators of protective immunity, but rather serve as surrogate markers [26,42].

### 4.4. Strengths and Limitations

This review has several strengths, namely, our meta-analysis was conducted on prospective studies, a robust design in observational studies. Furthermore, we conducted an extensive literature search without setting language restrictions, although only studies published in English were found, keeping in mind that some studies were excluded due to lack of relevant data and thereby others could have been missed. More importantly, this study provides further information regarding studies on cholera and immunity. The pooled estimates of serological antibodies provide insight for policymakers into planning future research on vaccines and improving the overall surveillance of cholera.

Although this is the first meta-analysis on serological antibodies after cholera, this study has some limitations. First, our pooled estimates were based on means and standard errors. Thus, our pooled estimates may be biased as sampling from individual studies was not obtained from the same population. However, this limitation was mitigated by the fact that all data from our quantitative analysis were reported from Bangladesh, and heterogeneity was not substantial as is evident in figures. Second, this meta-analysis demonstrated that only age was considered in the sensitivity analysis; disease susceptibility was not considered due to caveats in the data stratified by variables, such as nutritional status and blood group in primary studies. Future studies may need to take these factors into account.

Third, all participants were from Bangladesh (cholera-endemic country). Therefore, in countries where *V. cholerae* is not endemic, we are unable to determine the extent to which the pooled estimates of serological antibodies would vary in cholera convalescent patients as re-exposure to *V. cholerae* would be rare.

## 5. Conclusions

Seven cohort studies were analyzed to summarize changes in the serological antibody levels (vibriocidal, anti-CTB, and anti-LPS antibodies) over time after clinical cholera. Overall, our study strengthens previously published evidence that vibriocidal, CBT, and anti-LPS antibodies are approximately fourfold higher between days 7 and 30 compared to day 2 post-symptom onset. We also found strong evidence that these serological antibodies wane within 1 year following the symptoms onset. However, this decay should not be interpreted as waning immunity because immunity conferred through cholera against subsequent diseases lasts for 3–10 years [22,45]. This observation supports the hypothesis that longer-term protective immunity might be mediated by other immune markers (such as antibodies for O-specific polysaccharide) through anamnestic responses of memory B cells in the gut-associated lymphoid tissues. Our findings highlight the need for further studies to improve our understanding of immunity to *V. cholerae*. To the best of our knowledge, this is the first meta-analysis that combined data from cohort studies reporting the long-term kinetics of serological antibodies to *V. cholerae*, providing evidence to guide surveillance strategies and future research on vaccines. We advocate that serological cohort studies on cholera should also be performed in different populations (such as on African people), as *V. cholerae* strains may substantially vary in different geographical regions worldwide [6], and its epidemiology is different between African countries and Bangladesh [64]. As long as adequate drinking water is not available for all, infrastructure is not built, wars continue, and poverty prevails, cholera will remain a serious health issue in endemic areas.

## Figures and Tables

**Table 1:** Kinetics of vibriocidal antibody responses following cholera.

Days after Onset	Number of Studies (*n*)	Data Point (*n*)	Pooled Mean Vibriocidal Titer (95% CI)	Number of Studies (*n*)	Data Point (*n*)	Pooled Mean Vibriocidal Titer (95% CI) *
2	4	4	123.20 (77.00, 169.40)	5	6	123.00 (105.15, 140.85)
7	3	3	6955.94 (2444.88, 11,466.99)	4	5	4972.68 (2799.58, 7145.77)
30	4	4	2247.32 (1300.71, 3193.92)	5	6	1901.90 (1257.81, 2545.98)
90	4	4	578.60 (267.43, 889.78)	5	6	468.72 (272.63, 664.80)
180	3	3	345.88 (163.05, 525.72)	4	5	290.68 (190.39, 390.98)
360	3	3	177.18 (122.16, 232.21)	4	5	227.37 (177.18, 277.56) **

Definition of abbreviation: CI = confidence interval. * Sensitivity analysis including two studies with data from children aged ≤ 5 years. ** In two data points, sampling was performed on day 365.

**Table 2:** Kinetics of anti-cholera toxin B subunit IgA following cholera.

Days after Onset	Number of Studies (*n*)	Data Point (*n*)	Pooled Mean IgA Antibody Response to CTB (95% CI), ELISA Units	Number of Studies (*n*)	Data Point (*n*)	* Pooled Mean IgA Antibody Response to CTB (95% CI), ELISA Units *
2	6	7	5.32 (4.23, 6.41)	7	9	5.81 (4.70, 6.93)
7	4	5	49.07 (41.15, 56.99)	5	7	55.54 (46.51, 64.57)
30	6	7	20.88 (16.46, 25.30)	7	9	19.82 (15.63, 24.01)
90	6	7	9.86 (8.45, 11.27)	7	9	9.71 (7.83, 11.58)
180	5	6	8.30 (6.69, 9.91)	6	8	8.04 (6.12, 9.96)
360	3	3	6.40 (5.01, 7.78)	4	4	7.83 (5.05, 10.61) **

CTB, cholera toxin B subunit; CI, confidence interval * Sensitivity analysis including data from children aged ≤ 5 years. ** In two data points, sampling was carried out on day 365.

**Table 3:** Kinetics of anti-cholera toxin B subunit IgG following cholera.

Days after Onset	Number of Studies (*n*)	Data Point (*n*)	Pooled Mean IgG Antibody Response to CTB (95% CI), ELISA Units	Number of Studies (*n*)	Data Point (*n*)	Pooled Mean IgG Antibody Response to CTB (95% CI), ELISA Units *
2	6	7	17.66 (14.84, 20.48)	7	9	18.71 (16.02, 21.40)
7	4	5	64.64 (57.54, 71.75)	5	7	71.17 (62.41, 79.94)
30	6	7	68.62 (64.91, 72.32)	7	9	70.69 (66.81, 74.57)
90	6	7	42.18 (38.93, 45.42)	7	9	42.94 (40.03, 45.86)
180	5	6	29.27 (26.02, 32.51)	6	8	29.46 (26.75, 32.17)
360	3	3	23.52 (20.91, 26.14)	4	4	23.91 (22.77, 25.04) **

Definition of abbreviations: IgG, immunoglobulin G; CTB, cholera toxin B subunit; CI, confidence interval. * Sensitivity analysis including data from children aged ≤ 5 years. ** In two data points, sampling was performed on day 365.

**Table 4:** Kinetics of anti-lipopolysaccharide IgA following cholera.

Days after Onset	Number of Studies (*n*)	Data Point (*n*)	Pooled Mean IgA Antibody Response to LPS (95% CI), ELISA Units	Number of Studies (*n*)	Data Point (*n*)	Pooled Mean IgA Antibody Response to LPS (95% CI), ELISA Units *
2	5	6	9.43 (8.27, 10.58)	6	8	9.58 (8.86, 10.31)
7	3	4	124.46 (90.38, 158.54)	3	3	134.71 (91.74, 177.68)
30	5	6	43.94 (33.25, 54.63)	6	8	41.22 (29.47, 52.97)
90	5	6	17.40 (13.60, 21.19)	6	8	17.40 (14.72, 20.08)
180	5	6	12.74 (10.97, 14.51)	6	8	13.45 (11.55, 15.35)
360	3	3	10.22 (8.84, 11.60)	4	4	12.53 (11.37, 13.69) **

Definition of abbreviations: IgA, immunoglobulin A; LPS, lipopolysaccharide; CI, confidence interval. * Sensitivity analysis including data from children aged ≤ 5 years. ** In two data points, sampling was performed on day 365.

**Table 5:** Kinetics of anti-lipopolysaccharide IgG following cholera.

Days after Onset	Number of Studies (*n*)	Data Point (*n*)	Pooled Mean IgG Antibody Response to LPS (95% CI), ELISA Units	Number of Studies (*n*)	Data Point (*n*)	Pooled Mean IgG Antibody Response to LPS (95% CI), ELISA Units *
2	5	6	35.24 (32.88, 37.59)	6	8	35.88 (33.57, 38.18)
7	3	4	93.81 (80.47, 107.15)	3	3	101.75 (72.74, 130.76)
30	5	6	84.53 (76.24, 92.83)	6	8	86.29 (77.42, 95.16)
90	5	6	51.33 (37.44, 65.22)	6	8	53.57 (42.86, 64.28)
180	5	6	46.58 (35.54, 57.61)	6	8	48.40 (39.79, 57.00)
360	3	3	32.11 (25.04, 39.19)	4	4	34.34 (28.24, 40.43) **

Definition of abbreviations: IgG, immunoglobulin G; LPS, lipopolysaccharide; CI, confidence interval. * Sensitivity analysis including data from children aged ≤ 5 years. ** In two data points, sampling was performed on day 365.

## Data Availability

All relevant data are within the manuscript and its supporting information files.

## Supplementary Materials

The following supporting information can be downloaded at https://www.mdpi.com/article/10.3390/ijerph19127141/s1, Figure S1: Flow chart summarizing study evidence search and selection.

## Appendix A

**Table A1:** Characteristics of included studies *.

First Author, Year	Time Frame/Enrollment Dates	Country	Study Design	Sample Size and Participants	Immune Markers of Interest	Antibody Isotypes	Type of Blood Sample	*Vibrio cholerae* O1 Serotype	Measurement Methods	Multiple Time Points for Blood Collection	Follow-Up Duration (Days)
Azman, 2019 [35]	2006 to 2015 (Bangladesh); and September 2013 to September 2014 (USA)	Bangladesh and USA	Prospective cohort	38 North American volunteers (median age = 31; human challenge study) and 320 patients from Bangladesh (median age = 25)	Vibriocidal antibodies, and anti-CTB antibodies	IgA, IgG and IgM	Serum and plasma	Ogawa (*n* = 285); Inaba (*n* = 33)	ELISA	Day 0; 10; 28; 90; and 170 for volunteers; and day 2; 7; 30; 90; 180, 270, 365, 540, 720, and 900 for patients	915
Hossain, 2019 [36]	September 2013 to September 2014 (USA)	USA and Bangladesh	Prospective cohort	38 North America volunteers (median age = 31; human challenge study) and 38 patients from Bangladesh (median age = 30)	OSP-specific antibodies	IgA, IgG and IgM	Serum and plasma	Inaba (*n* = 80) for patients	ELISA	Day 0; 10; 28; 90; and 170 for volunteers; and day 2; 7; 21 or 30; 90; 180 for patients	170 to 180
Aktar, 2016 [37]	February 2012 to April 2014	Bangladesh	Prospective cohort	Cholera patients; *n* = 60 (2–5 y, *n* = 11; 6–17 y, *n* = 21; and 18–55 y, *n* = 28)	Vibriocidal antibodies; anti-CTB and anti-LPS antibodies	IgA and IgG	Plasma	Ogawa only	Guinea pig complement; ELISA	Days 2; 7; 30; 90; and 180	180
Alam, 2013 [38]	December 2006 to May 2008	Bangladesh	Prospective cohort	30 (median age: 31)	Anti-CTB and LPS antibodies	IgA and IgG	Plasma	Ogawa (*n* = 20); Inaba (*n* = 10)	ELISA	Day 30; 90; 180; 270; and 360	360
Alam, 2011 [39]	October 2008 and June 2010	Bangladesh	Prospective cohort	Cholera patients (*n* = 70; adult)	Vibriocidal antibodies, anti-CTB and anti-LPS antibodies	IgA and IgG	Plasma	Ogawa (*n* = 55) and Inaba (15)	ELISA; ELISA	Day 3; 30; 90; 180; 270; and 360	360
Uddin, 2011 [40]	Not reported	Bangladesh	Prospective cohort	18; Patients had severe cholera. Median age = 30	Anti-CTB and LPS antibodies	IgA and IgG	Duodenal biopsy and plasma	Ogawa (*n* = 16); Inaba (*n* = 2)	ELISA	Day 2; 7; 30; 90; 180; and 360	360
Kendall, 2010 [41]	April 2007 to April 2009	Bangladesh	Prospective cohort	*n* = 41 (26 of these were frozen samples) Mean age = 30	Vibriocidal antibodies; anti-CTB and anti-LPS antibodies	IgA, IgG, and IgM	Plasma	Ogawa (*n* = 32); Inaba (*n* = 9)	ELISA	Day 2; 7; 30; 90	90
Harris, 2009 [42]	December 2006 to May 2008	Bangladesh	Prospective cohort	Cholera patients (*n* = 39; median age: 24 y)	Vibriocidal antibodies, TcpA, anti-CTB, ASC and LPS responses	IgA and IgG	Serum and plasma	Ogawa (*n* = 26) and Inaba (*n* = 13)	Guinea pig complement; ELISA	Day 2; 7; 30; 90; 180; 270; and 360	360
Jayasekera, 2008 [43]	December 2006 to May 2007	Bangladesh	Prospective cohort	14 (mean ag: 30). Patients had severe cholera	Vibriocidal antibodies; anti-CTB and anti-LPS antibodies	IgA and IgG	Serum	Ogawa (*n* = 7); Inaba (*n* = 7)	ELISA	Day 2; 7; 30; 90	90

ELISA, enzyme-linked immunosorbent assay; y, years; CTB, cholera toxin B subunit; LPS, lipopolysaccharide; OSP, O-specific polysaccharide. * In some studies, other immune markers such as memory B cells were also evaluated.

**Table A2:** Quality assessment of included studies (*n* = 9) #.

Risk of Bias Assessment				
Author, Year, Reference	Selection of Participants (4 Stars Could Be Awarded)	Comparability (2 Stars Could Be Awarded)	Outcome (3 Stars Could Be Awarded)	Total Score (A Maximum of 9 Stars Could Be Awarded)
	Representativeness of the Exposed Cohort; Selection of Controls; Ascertainment of Exposure; Demonstration That Outcome of Interest Was Not Present at the Start of the Study	Comparability of Cohorts on the Basis of the Design or Analysis; Additional Factors	Assessment of Outcome; Adequate Follow-Up Period for Outcome of Interest to Occur; Complete Follow-Up (All Subjects Accounted for)	
Azman, 2019 [35]	***	**	***	8
Hossain, 2019 [36]	***	**	***	8
Aktar, 2016 [37]	***	*	**	6
Alam, 2013 [38]	****	**	***	9
Alam, 2011 [39]	***	*	**	6
Uddin, 2011 [40]	**	**	***	7
Kendall, 2010 [41]	***	*	**	6
Harris, 2009 [42]	***	**	***	8
Jayasekera, 2008 [43]	**	**	**	6

# The quality of the studies was assessed using the Newcastle-Ottawa Scale for cohort studies [33]. A star is assigned to each study for each reported item to facilitate a rapid visual assessment. A study with the highest quality could be awarded up to 9 stars [33].

## References

[1] World Health Organization (WHO) Cholera. https://www.who.int/health-topics/cholera#tab=tab_1.

[2] Kanungo S., Azman A.S., Ramamurthy T., Deen J., Dutta S. (2022). Cholera. Lancet.

[3] World Health Organization (WHO) (2017). Cholera vaccines: WHO position paper—August 2017. Wkly. Epidemiol. Rec..

[4] Lipp E.K., Huq A., Colwell R.R. (2002). Effects of global climate on infectious disease: The cholera model. Clin. Microbiol. Rev..

[5] Alam M., Sultana M., Nair G.B., Siddique A.K., Hasan N.A., Sack R.B., Sack D.A., Ahmed K.U., Sadique A., Watanabe H. (2007). Viable but nonculturable *Vibrio cholerae* O1 in biofilms in the aquatic environment and their role in cholera transmission. Proc. Natl. Acad. Sci. USA.

[6] Muzembo B.A., Kitahara K., Ohno A., Debnath A., Okamoto K., Miyoshi S.-I. (2021). Cholera rapid diagnostic tests for the detection of *Vibrio cholerae* O1: An updated meta-analysis. Diagnostics.

[7] Yoon S.H., Waters C.M. (2019). *Vibrio* *cholerae*. Trends Microbiol..

[8] Clemens J.D., Nair G.B., Ahmed T., Qadri F., Holmgren J. (2017). Cholera. Lancet.

[9] Hu D., Liu B., Feng L., Ding P., Guo X., Wang M., Cao B., Reeves P.R., Wang L. (2016). Origins of the current seventh cholera pandemic. Proc. Natl. Acad. Sci. USA.

[10] Leung D.T., Chowdhury F., Calderwood S.B., Qadri F., Ryan E.T. (2012). Immune responses to cholera in children. Expert Rev. Anti-Infect. Ther..

[11] Legros D. (2018). Partners of the global task force on cholera control global cholera epidemiology: Opportunities to reduce the burden of cholera by 2030. J. Infect. Dis..

[12] Muzembo B.A., Kitahara K., Debnath A., Ohno A., Okamoto K., Miyoshi S.-I. (2022). Cholera outbreaks in India, 2011–2020: A systematic review. Int. J. Environ. Res. Public Health.

[13] Goma Epidemiology Group (1995). Public health impact of Rwandan refugee crisis: What happened in Goma, Zaire, in July 1994?. Lancet.

[14] Camacho A., Bouhenia M., Alyusfi R., Alkohlani A., Naji M.A.M., de Radiguès X., Abubakar A.M., Almoalmi A., Seguin C., Sagrado M.J. (2018). Cholera epidemic in Yemen, 2016–2018: An analysis of surveillance data. Lancet Glob. Health.

[15] Ingelbeen B., Hendrickx D., Miwanda B., Van Der Sande M.A., Mossoko M., Vochten H., Riems B., Nyakio J.-P., Vanlerberghe V., Lunguya O. (2019). Recurrent cholera outbreaks, democratic republic of the Congo, 2008–2017. Emerg. Infect. Dis..

[16] Centers for Disease Control and Prevention (CDC) (2010). Cholera outbreak—Haiti, October 2010. MMWR Morb. Mortal. Wkly. Rep..

[17] Ali M., Nelson A., Lopez A.L., Sack D.A. (2015). Updated global burden of cholera in endemic countries. PLoS Negl. Trop. Dis..

[18] Barzilay E.J., Schaad N., Magloire R., Mung K.S., Boncy J., Dahourou G.A., Mintz E.D., Steenland M.W., Vertefeuille J.F., Tappero J.W. (2013). Cholera surveillance during the haiti epidemic—The first 2 Years. N. Engl. J. Med..

[19] World Health Organization (WHO) (2009). Cholera outbreak, Zimbabwe. Wkly. Epidemiol. Rec..

[20] Azman A.S., Luquero F.J., Ciglenecki I., Grais R.F., Sack D.A., Lessler J. (2015). The impact of a one-dose versus two-dose oral cholera vaccine regimen in outbreak settings: A modeling study. PLoS Med..

[21] Teoh S.L., Kotirum S., Hutubessy R.C.W., Chaiyakunapruk N. (2017). Global economic evaluation of oral cholera vaccine: A systematic review. Hum. Vaccines Immunother..

[22] Ali M., Emch M., Park J.K., Yunus M., Clemens J. (2011). Natural cholera infection-derived immunity in an endemic setting. J. Infect. Dis..

[23] Pasetti M.F., Levine M.M. (2012). Insights from natural infection-derived immunity to cholera instruct vaccine efforts. Clin. Vaccine Immunol..

[24] Metcalf C.J.E., Farrar J., Cutts F.T., Basta N.E., Graham A.L., Lessler J., Ferguson N.M., Burke D.S., Grenfell B.T. (2016). Use of serological surveys to generate key insights into the changing global landscape of infectious disease. Lancet.

[25] Leung T., Matrajt L. (2021). Protection afforded by previous *Vibrio cholerae* infection against subsequent disease and infection: A review. PLoS Negl. Trop. Dis..

[26] Patel S.M., Rahman M.A., Mohasin M., Riyadh M.A., Leung D., Alam M.M., Chowdhury F., Khan A.I., Weil A.A., Aktar A. (2012). Memory B cell responses to *Vibrio cholerae* O1 lipopolysaccharide are associated with protection against infection from household contacts of patients with cholera in Bangladesh. Clin. Vaccine Immunol..

[27] Mukhopadhyay S., Nandi B., Ghose A. (2000). Antibodies (IgG) to lipopolysaccharide of *Vibrio cholerae* O1 mediate protection through inhibition of intestinal adherence and colonisation in a mouse model. FEMS Microbiol. Lett..

[28] Ryan E.T., Leung D.T., Jensen O., Weil A.A., Bhuiyan T.R., Khan A.I., Chowdhury F., LaRocque R.C., Harris J.B., Calderwood S.B. (2021). Systemic, mucosal, and memory immune responses following cholera. Trop. Med. Infect. Dis..

[29] Benenson A.S., Saad A., Paul M. (1968). Serological studies in cholera. I. Vibrio agglutinin response of cholera patients determined by a microtechnique. Bull. World Health Organ..

[30] Harris J.B., Larocque R.C., Chowdhury F., Khan A.I., Logvinenko T., Faruque A.S.G., Ryan E.T., Qadri F., Calderwood S.B. (2008). Susceptibility to *Vibrio cholerae* infection in a cohort of household contacts of patients with cholera in Bangladesh. PLoS Negl. Trop. Dis..

[31] Harris J.B. (2018). Cholera: Immunity and prospects in vaccine development. J. Infect. Dis..

[32] Moher D., Liberati A., Tetzlaff J., Altman D.G., PRISMA Group (2009). Preferred reporting items for systematic reviews and meta-analyses: The PRISMA statement. Ann. Intern. Med..

[33] Wells G.A., Shea B., O’Connell D., Pereson J., Welch V., Losos M., Tugwell P. The Newcastle–Ottawa Scale (NOS) for Assessing the Quality of Nonrandomised Studies in Meta-Analyses. http://www.ohri.ca/programs/clinical_epidemiology/oxford.asp.

[34] Higgins J.P.T., Thompson S.G. (2002). Quantifying heterogeneity in a meta-analysis. Stat. Med..

[35] Azman A.S., Lessler J., Luquero F.J., Bhuiyan T.R., Khan A.I., Chowdhury F., Kabir A., Gurwith M., Weil A.A., Harris J.B. (2019). Estimating cholera incidence with cross-sectional serology. Sci. Transl. Med..

[36] Hossain M., Islam K., Kelly M., Mayo Smith L.M., Charles R.C., Weil A.A., Bhuiyan T.R., Kováč P., Xu P., Calderwood S.B. (2019). Immune responses to O-specific polysaccharide (OSP) in North American adults infected with *Vibrio cholerae* O1 Inaba. PLoS Negl. Trop. Dis..

[37] Aktar A., Rahman M.A., Afrin S., Faruk M.O., Uddin T., Akter A., Sami M.I.N., Yasmin T., Chowdhury F., Khan A.I. (2016). O-specific polysaccharide-specific memory B cell responses in young children, older children, and adults infected with *Vibrio cholerae* O1 Ogawa in Bangladesh. Clin. Vaccine Immunol..

[38] Alam M.M., Arifuzzaman M., Ahmad S.M., Hosen M.I., Rahman M.A., Rashu R., Sheikh A., Ryan E.T., Calderwood S.B., Qadri F. (2013). Study of avidity of antigen-specific antibody as a means of understanding development of long-term immunological memory after *Vibrio cholerae* O1 infection. Clin. Vaccine Immunol..

[39] Alam M.M., Riyadh M.A., Fatema K., Rahman M.A., Akhtar N., Ahmed T., Chowdhury M.I., Chowdhury F., Calderwood S.B., Harris J.B. (2011). Antigen-specific memory B-cell responses in Bangladeshi adults after one- or two-dose oral killed cholera vaccination and comparison with responses in patients with naturally acquired cholera. Clin. Vaccine Immunol..

[40] Uddin T., Harris J.B., Bhuiyan T.R., Shirin T., Uddin M.I., Khan A.I., Chowdhury F., LaRocque R.C., Alam N.H., Ryan E.T. (2011). Mucosal immunologic responses in cholera patients in Bangladesh. Clin. Vaccine Immunol..

[41] Kendall E.A., Tarique A.A., Hossain A., Alam M.M., Arifuzzaman M., Akhtar N., Chowdhury F., Khan A.I., LaRocque R.C., Harris J.B. (2010). Development of immunoglobulin M memory to both a T-cell-independent and a T-cell-dependent antigen following infection with *Vibrio cholerae* O1 in Bangladesh. Infect. Immun..

[42] Harris A.M., Bhuiyan M.S., Chowdhury F., Khan A.I., Hossain A., Kendall E.A., Rahman A., LaRocque R.C., Wrammert J., Ryan E.T. (2009). Antigen-specific memory B-cell responses to *Vibrio cholerae* O1 infection in Bangladesh. Infect. Immun..

[43] Jayasekera C.R., Harris J.B., Bhuiyan S., Chowdhury F., Khan A.I., Faruque A.S.G., Larocque R.C., Ryan E.T., Ahmed R., Qadri F. (2008). Cholera toxin–specific memory B cell responses are induced in patients with dehydrating diarrhea caused by *Vibrio cholerae* O1. J. Infect. Dis..

[44] Charles R.C., Kelly M., Tam J.M., Akter A., Hossain M., Islam K., Biswas R., Kamruzzaman M., Chowdhury F., Khan A.I. (2020). Humans surviving cholera develop antibodies against *Vibrio cholerae* O-specific polysaccharide that inhibit pathogen motility. mBio.

[45] Levine M.M., Black R., Clements M.L., Cisneros L., Nalin D.R., Young C.R. (1981). Duration of infection-derived immunity to cholera. J. Infect. Dis..

[46] Holmgren J. (2021). An update on cholera immunity and current and future cholera vaccines. Trop. Med. Infect. Dis..

[47] Iyer A.S., Harris J.B. (2021). Correlates of protection for cholera. J. Infect. Dis..

[48] Johnson R.A., Uddin T., Aktar A., Mohasin M., Alam M.M., Chowdhury F., Harris J.B., Larocque R.C., Bufano M.K., Yu Y. (2012). Comparison of immune responses to the O-specific polysaccharide and lipopolysaccharide of *Vibrio cholerae* O1 in Bangladeshi adult patients with cholera. Clin. Vaccine Immunol..

[49] Desai S.N., Cravioto A., Sur D., Kanungo S. (2014). Maximizing protection from use of oral cholera vaccines in developing country settings: An immunological review of oral cholera vaccines. Hum. Vaccines Immunother..

[50] Simanjuntak C.H., O’Hanley P., Punjabi N.H., Noriega F., Pazzaglia G., Dykstra P., Kay B., Budiarso A., Rifai A.R., Suharyono (1993). Safety, immunogenicity, and transmissibility of single-dose live oral cholera vaccine strain CVD l03-HgR in 24- to 59-month-old Indonesian children. J. Infect. Dis..

[51] Haney D.J., Lock M.D., Gurwith M., Simon J.K., Ishioka G., Cohen M.B., Kirkpatrick B.D., Lyon C.E., Chen W.H., Sztein M.B. (2018). Lipopolysaccharide-specific memory B cell responses to an attenuated live cholera vaccine are associated with protection against *Vibrio cholerae* infection. Vaccine.

[52] Weil A.A., Arifuzzaman M., Bhuiyan T.R., LaRocque R.C., Harris A.M., Kendall E.A., Hossain A., Tarique A.A., Sheikh A., Chowdhury F. (2009). Memory T-Cell responses to *Vibrio cholerae* O1 infection. Infect. Immun..

[53] Richie E.E., Punjabi N.H., Sidharta Y.Y., Peetosutan K.K., Sukandar M.M., Wasserman S.S., Lesmana M.M., Wangsasaputra F.F., Pandam S.S., Levine M.M. (2000). Efficacy trial of single-dose live oral cholera vaccine CVD 103-HgR in North Jakarta, Indonesia, a cholera-endemic area. Vaccine.

[54] McCarty J., Bedell L., de Lame P.-A., Cassie D., Lock M., Bennett S., Haney D. (2021). Update on CVD 103-HgR single-dose, live oral cholera vaccine. Expert Rev. Vaccines.

[55] Weil A.A., Chowdhury F., Khan A.I., Leung D.T., Uddin T., Begum Y.A., Saha N.C., Charles R.C., Larocque R.C., Harris J.B. (2012). Frequency of reexposure to *Vibrio cholerae* O1 evaluated by subsequent vibriocidal titer rise after an episode of severe cholera in a highly endemic area in Bangladesh. Am. J. Trop. Med. Hyg..

[56] Mosley W.H., Ahmad S., Benenson A.S., Ahmed A. (1968). The relationship of vibriocidal antibody titre to susceptibility to cholera in family contacts of cholera patients. Bull. World Health Organ..

[57] Ritter A.S., Chowdhury F., Franke M.F., Becker R.L., Bhuiyan T.R., Khan A.I., Saha N.C., Ryan E.T., Calderwood S.B., Larocque R.C. (2019). Vibriocidal titer and protection from cholera in children. Open Forum Infect. Dis..

[58] Glass R.I., Svennerholm A.-M., Khan M.R., Huda S., Huq M.I., Holmgren J. (1985). Seroepidemiological studies of EI tor cholera in Bangladesh: Association of serum antibody levels with protection. J. Infect. Dis..

[59] Okuno T., Kashige N., Satho T., Irie K., Hiramatsu Y., Sharmin T., Fukumitsu Y., Uyeda S., Yamada S., Harakuni T. (2013). Expression and secretion of cholera toxin B subunit in lactobacilli. Biol. Pharm. Bull..

[60] Baldauf K.J., Royal J., Hamorsky K.T., Matoba N. (2015). Cholera toxin B: One subunit with many pharmaceutical applications. Toxins.

[61] Clemens J.D., Sack D.A., Harris J.R., Van Loon F., Chakraborty J., Ahmed F., Rao M.R., Khan M.R., Yunus M.D., Huda N. (1990). Field trial of oral cholera vaccines in Bangladesh: Results from three-year follow-up. Lancet.

[62] Yuki Y., Nojima M., Hosono O., Tanaka H., Kimura Y., Satoh T., Imoto S., Uematsu S., Kurokawa S., Kashima K. (2021). Oral MucoRice-CTB vaccine for safety and microbiota-dependent immunogenicity in humans: A phase 1 randomised trial. Lancet Microbe.

[63] Aktar A., Rahman M.A., Afrin S., Akter A., Uddin T., Yasmin T., Sami I.N., Dash P., Jahan S.R., Chowdhury F. (2018). Plasma and memory B cell responses targeting O-specific polysaccharide (OSP) are associated with protection against *Vibrio cholerae* O1 infection among household contacts of cholera patients in Bangladesh. PLoS Negl. Trop. Dis..

[64] Sack D.A., Debes A.K., Ateudjieu J., Bwire G., Ali M., Ngwa M.C., Mwaba J., Chilengi R., Orach C.C., Boru W. (2021). Contrasting epidemiology of cholera in Bangladesh and Africa. J. Infect. Dis..

