# Supplementary figures and images for: Long-Term Kinetics of Serological Antibodies against Vibrio cholerae Following a Clinical Cholera Case: A Systematic Review and Meta-Analysis
Source: Int J Environ Res Public Health. 2022 Jun 10;19(12):7141. doi: 10.3390/ijerph19127141 (PMC9223532; doi:10.3390/ijerph19127141)

## Supplementary Figure

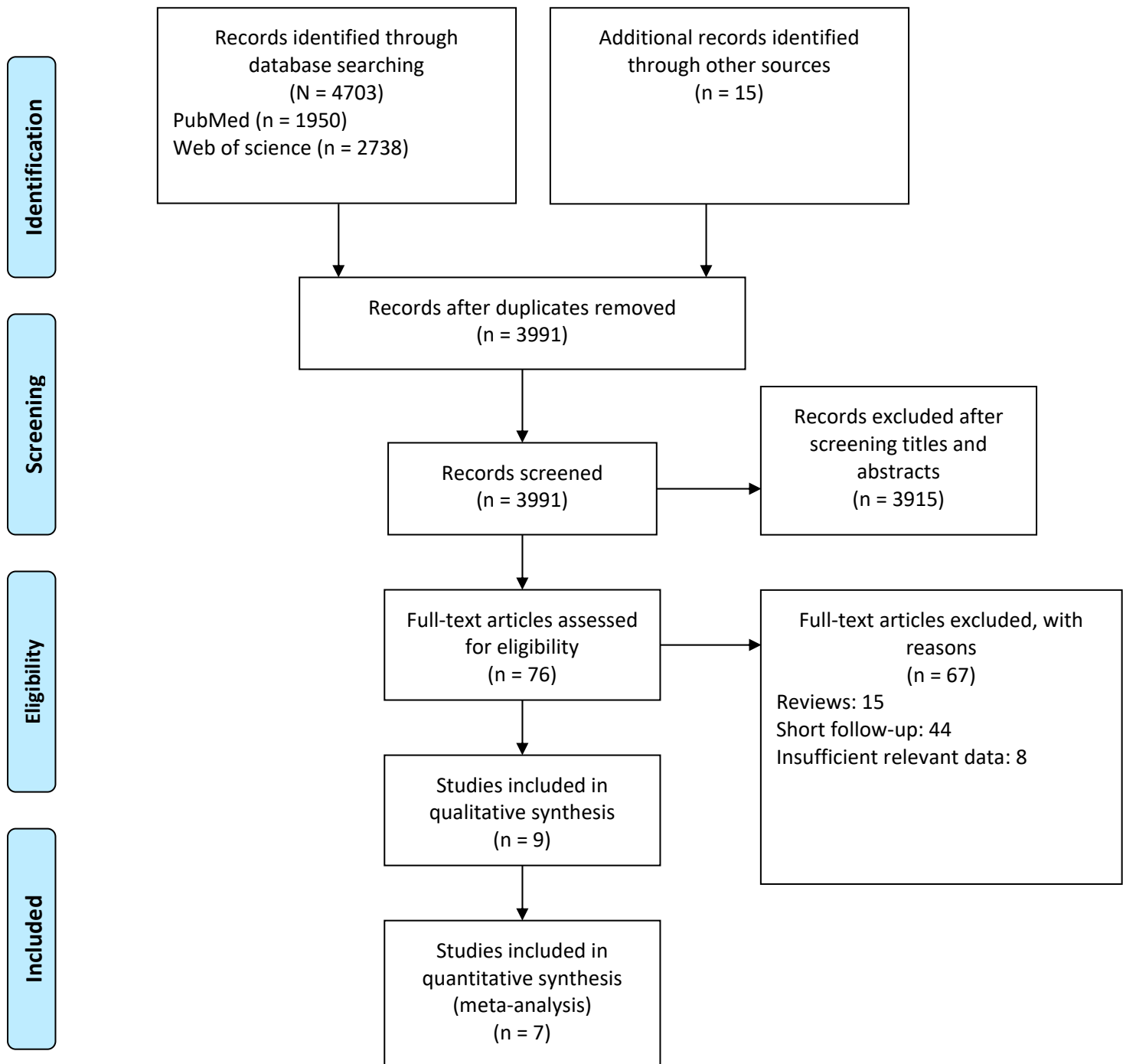

**Figure S1.** Flow chart summarizing study evidence search and selection.

Supplement: Supplementary file 1 [file ijerph-19-07141-s001.zip › ijerph-1732425-supplementary.pdf]
